# Supplementary material for: Bioinformatics-Guided Identification of Ethyl Acetate Extract of Citri Reticulatae Pericarpium as a Functional Food Ingredient with Anti-Inflammatory Potential
Source: Molecules. 2022 Aug 25;27(17):5435. doi: 10.3390/molecules27175435 (PMC9457579; doi:10.3390/molecules27175435)
Supplement: Supplementary file 1 [file molecules-27-05435-s001.zip › molecules-1860714-Tables S1-S4.pdf]

Table S1 Clusters identified by principal compoenent analysis and K-means clustering

PC1	PC2	PC3	Name	Type	Cluster in K-means	Labels used in Figure 2
-1.098	-0.719	0.415	Hesperitin	Flavone and Flavonoids	0	I
-2.079	-0.325	0.34	Naringenin	Flavone and Flavonoids	0	I
-1.301	-0.892	0.195	5,7-dihydroxy-2-(3-hydroxy-4-methoxyphenyl)chroman-4-one	Flavone and Flavonoids	0	I
-1.807	-0.395	0.269	Quercetin	Flavone and Flavonoids	0	I
-0.047	-1.151	-0.185	Gardenin_B	Polymethoxy flavone	0	I
0.746	-1.456	-0.237	Tangeretin	Polymethoxy flavone	0	I
0.471	-1.294	-0.261	Nobiletin	Polymethoxy flavone	0	I
-0.268	-1.382	0.2	Carvacrol	Aromatic	0	I
-0.137	-0.465	0.374	Vanillin	Aromatic	0	I
1.483	-0.451	0.675	5-hydroxymethyl furfural	Aromatic	0	I
0.663	-0.165	0.47	Caffeic acid	Aromatic	0	I
-0.249	-0.21	-0.242	Isovanillic	Aromatic	0	I
0.229	-1.15	-0.098	Antioxidant No. 33	Aromatic	0	I
1.109	-1.093	0.351	p-Cymen-8-ol	Aromatic	0	I
1.066	-0.926	0.566	Dimethyl anthranilate	Aromatic	0	I
-0.482	-0.461	-0.331	4-acetylbenzoic	Aromatic	0	I
1.124	-1.714	0.565	Delta-amorphene	Sesquiterpenoids	0	I
1.783	-1.85	0.679	()-Cuparene	Sesquiterpenoids	0	I
1.66	-1.989	0.688	()-Ledene	Sesquiterpenoids	0	I
1.896	-2.042	0.617	Germacrene A	Sesquiterpenoids	0	I
1.197	-1.263	0.462	(R)-p-Menth-1-en-4-ol	Monoterpenoids	0	I
1.603	-1.216	0.537	Alpha-thujene	Monoterpenoids	0	I
1.293	-1.04	0.272	2-(2-butynyl)-cyclohexanone	Monoterpenoids	0	I
-2.005	3.894	0.212	Hesperidin	Flavone glycosides	3	II
-1.55	1.72	-1.934	Neohesperdin	Flavone glycosides	3	II
-1.392	1.657	-2.046	Naringin	Flavone glycosides	3	II
0.116	2.89	0.107	Neohesperidin	Flavone glycosides	3	II
4.185	2	0.568	Heptamethoxyflavone	Polymethoxy flavone	2	III
3.715	0.115	-1.594	Citromitin	Polymethoxy flavone	2	III
1.649	0.397	2.32	Sinensetin	Polymethoxy flavone	2	III
4.685	-2.064	1.205	Diisobutyl phthalate	Aromatic	2	III
2.247	-1.463	0.764	Diethyl phthalate	Aromatic	2	III
1.93	-1.419	0.707	(L)-alpha-Terpineol	Monoterpenoids	2	III
2.835	-2.009	0.824	(-)-alpha-Pinene	Monoterpenoids	2	III
2.139	-1.534	0.808	()-2-Carene	Monoterpenoids	2	III
4.368	-1.343	1.036	(R)-linalool	Monoterpenoids	2	III
5.364	-1.681	1.485	Beta-Ocimene	Monoterpenoids	2	III
1.831	-1.299	0.681	(-)-Sabinene	Monoterpenoids	2	III
1.231	-1.323	0.424	L-4-terpineol	Monoterpenoids	2	III
2.008	-1.222	0.618	Beta-terpineol	Monoterpenoids	2	III
3.917	-1.339	0.859	22410-74-8	Monoterpenoids	2	III
4.655	-1.62	1.174	Neral	Fatty	2	III
5.417	-1.729	1.517	cis-beta-Ocimene	Fatty	2	III
4.976	-1.778	1.343	3-decyn-2-ol	Fatty	2	III
5.343	-1.913	1.495	cis-2,6-Dimethyl-2,6-octadiene	Fatty	2	III
4.811	1.01	0.921	Sitosterol	Others	2	III
3.371	-1.861	0.777	Neryl acetate	Others	2	III
5.443	-1.565	1.375	(2S)-2-ethoxypentane	Others	2	III

4.812	-1.118	1.347	Eufin	Others	2	III
6.728	-2.131	1.931	2,5,5-trimethylhepta-1,6-diene	Monoterpenoids	1	IV
9.901	-1.981	2.444	Undecanal	Fatty	1	IV
9.14	-2.162	2.109	lauric acid	Fatty	1	IV
6.438	-1.951	1.458	Octanal	Fatty	1	IV
8.471	-2.354	1.515	Stearic acid	Fatty	1	IV
9.223	-2.851	1.734	Henicosane	Fatty	1	IV
6.669	-2.106	1.821	alpha-Ocimene	Fatty	1	IV
8.569	-2.787	2.069	Farnesane	Fatty	1	IV
9.944	-1.907	2.54	1-Undecyne	Fatty	1	IV
9.874	-2.039	2.503	Dodec-2-enal	Fatty	1	IV
9.896	-1.896	2.536	1-Decanol	Fatty	1	IV
7.326	-2.165	1.756	Alpha-sinensal	Fatty	1	IV
8.518	-2.55	1.956	2,6,11-trimethyldodecane	Fatty	1	IV
8.439	-2.02	2.042	6-hepten-1-ol	Fatty	1	IV
9.507	-2.499	2.114	Undecyl acetate	Fatty	1	IV
8.125	-2.503	1.864	(+)-Citronellyl butyrate	Others	1	IV
6.565	-1.679	1.882	Isoprenol	Others	1	IV

Table S2 Physiochemical properties of compounds included in Cluster I

Name	Oral Bioavailability	MW	AlogP or logP	caco2	BBB
Naringenin	59.29	272.27	2.30	0.28	-0.37
5,7-dihydroxy-2-(3-hydroxy-4-methoxyphenyl)chroman-4-one	47.74	302.30	2.28	0.28	-0.30
Tangeretin	21.38	372.40	3.06	1.23	0.09
Nobiletin	61.67	402.43	3.04	1.05	-0.08
Carvacrol	46.69	136.26	2.87	1.89	2.27
Vanillin	52.00	152.16	1.31	0.68	0.41
Caffeic acid	28.66	164.17	1.17	0.33	-0.17
Isovanillic	39.42	168.16	1.15	0.47	0.38
Antioxidant No. 33	26.74	206.36	4.36	1.68	1.81
p-Cymen-8-ol	32.26	150.24	2.29	1.33	1.35
Dimethyl anthranilate	65.87	165.21	1.49	1.35	1.29
4-acetylbenzoic	28.66	164.17	1.17	0.33	-0.17
Delta-amorphene	17.95	204.39	4.94	1.85	1.99
()-Cuparene	38.26	202.37	4.72	1.88	2.15
()-Ledene	51.84	204.39	4.36	1.86	2.16
Germacrene A	19.21	204.39	5.33	1.88	2.11
(R)-p-Menth-1-en-4-ol	32.16	154.28	2.55	1.33	1.52
Alpha-thujene	46.25	136.26	2.87	1.85	2.30
2-(2-butynyl)-cyclohexanone	47.78	150.24	2.68	1.44	1.48

Table S3 Compound identifed in HPLC/MS/MS analysis

No.	t_R (min)	Formula	[M+H]			[M-H]			Compound	Ref	Peak areas			Ab. in EtOAC
			Observed	Theoretical	Fragments	Observed	Theoretical	Fragments			EtOAc	EtOH	Ratio	
1	3.11	$C_9H_7O_4$				179.0343	179.0344	135.045, 134.0367, 117.0499, 90.9981	caffeic acid	[1]	16352	N.D	>10	↑
2	3.36	$C_{27}H_{30}O_{15}$	595.1669	595.1663		593.1509	593.1466	503.1183, 473.1080, 383.0736, 353.0656	vicenin-2	[2]	N.D	23599	<0.1	↓
3	3.86	$C_{28}H_{32}O_{16}$				623.1619	623.1612	533.1282, 503.1191, 413.0872, 383.0768	stellarin-2	[2]	3757	29986	0.06	↓
4	5.00	$C_{10}H_{10}O_4$				193.0502	193.0501	178.0263, 134.0363, 106.0414	ferulic acid	[2]	24541	5329	2.07	↑
5	5.50	$C_{22}H_{22}O_{11}$	463.1246	463.124	379.0739, 313.0703, 343.0815, 367.0799, 353.1032, 409.0940, 427.1033	461.1079	481.1084	371.0756, 341.0656, 298.0471	diosmetin-8-C-glucoside	[2]	25980	28010	0.42	↔
6	5.90	$C_{27}H_{31}O_{14}$				579.1718	579.1714	271.0606, 151.0024	naringin	[2]	5897	26255	0.10	↓

7	6.47	$C_{28}H_{34}O_{15}$				609.1827	609.1819	301.0716, 257.0806, 242.0570, 199.0389, 151.0031	neohesperidin	[2]	362320	477320	0.34	↓
8	7.84	$C_{29}H_{32}O_{16}$	637.1763	637.1769	389.1224, 374.1027, 359.0673				hexamethoxyflavone malonyl glucoside	[2]	1549	3930	0.18	↓
9	8.33	$C_{26}H_{30}O_{10}$				501.1771	51.1761	N.D	shihulimonin	[2]	150447	5549	12.22	↑
10	8.56	$C_{26}H_{30}O_{10}$				501.1759	51.1761	N.D	limonexic acid	[2]	69931	13498	2.33	↑
11	8.85	$C_{18}H_{16}O_{6}$	329.1031	329.1025	314.0789, 299.0559				hydroxy-trimethoxy flavone	[2]	16869	3612	2.10	↑
12	10.21	$C_{16}H_{14}O_{6}$				307.0712	307.0712	286.0480, 242.0578, 199.0389, 164.0098	hesperetin	[2]	72817	N.D	>10	↑
13	11.35	$C_{20}H_{20}O_{8}$	389.1238	389.1236	373.1292, 358.1047, 343.0821, 329.1039				isosinensetin	[2]	348509	9650	16.27	↑
14	11.38	$C_{19}H_{18}O_{7}$				357.0979	357.0974	N.D	5-desmethylsinensetin	[2]	121394	18336	2.98	↔

15	11.80	$C_{20}H_{20}O_8$				387.1076	387.108	372.0842,<br>357.0605,<br>342.0359,<br>329.0667	umuhengerin	[2]	36866	3435	4.84	↑
16	12.89	$C_{20}H_{20}O_8$	389.1238	389.1236	373.1288,<br>357.0967,<br>343.0818,<br>329.1026,<br>312.1000				sinensetin	[2]	459971	122211	1.70	↔
17	13.32	$C_{19}H_{18}O_6$	343.1187	343.1182	328.0945,<br>313.0716,<br>299.0930,<br>285.0755				5,6,7,4′-tetramethoxyflavone	[2]	567831	151794	1.69	↔
18	14.67	$C_{18}H_{16}O_7$				343.082	343.0818	343.0814,<br>328.0580,<br>313.0353,<br>298.0108	xanthomicrol	[2]	75723	7386	4.62	↑
19	14.98	$C_{20}H_{20}O_7$	403.1394	403.1393	373.0926,<br>355.0819,<br>327.0871,<br>342.1102				nobiletin	[2]	2486978	1367362	0.82	↑
20	15.23	$C_{19}H_{18}O_6$	343.1192	343.1182	328.0949,<br>313.0722,<br>310.0846,<br>299.0930,<br>282.0896				5,6,7,4′-tetramethoxyflavone	[2]	2705519	1360418	0.90	↔

21	15.37	$C_{19}H_{18}O_8$				373.093	373.0923	343.0450, 358.0684, 328.0208	5,6-Dihydroxy-7,8,3′,4′-tetramethoxyflavone	[3]	193235	28354	3.07	↑
22	16.42	$C_{22}H_{24}O_9$	433.1501	433.1499	418.1252, 403.1022, 385.0911, 357.0900				3-Methoxynobiletin	[4]	120279	15431	3.51	↑
23	17.54	$C_{20}H_{20}O_7$	373.1287	373.1287	343.0821, 358.1028, 325.0705, 297.0743				tangeretin	[2]	2705519	1360418	0.90	↔
24	19.29	$C_{20}H_{20}O_8$	389.1248	389.1236	374.1002, 359.0775, 341.0666, 328.0948				5-Demethylnobiletin	[2]	994658	179672	2.49	↑
25	22.32	$C_{19}H_{18}O_7$	359.1136	359.1131	359.1137, 344.0889, 329.0673, 311.0561, 298.0827				Gardenin B	[4]	77581	5193	6.73	↑
Peak Area of identified compounds											1.16E+07	5.25E+06		

Table S4 Results from re-docking

Name	PDBID	RMSD versus co-crystal ligand
AKT1	4EJN	0.738
PI3K	3L54	0.952
JNK	3ELJ	0.722
PDE3A	7L29	2.089
NOS2	4NOS	0.327
NOS1	6CIC	1.534
SYK	1XBC	0.592
STAT1	1BF5	Not applicable
STAT3	1BG1	Not applicable
STAT5	1Y1U	Not applicable
cSRC	2BDF	1.797
PPARG	3GZ9	0.529
TLR4	3VQ1	Not applicable
MAPK14	1A9U	0.879
MTOR	4JT6	0.735
ITGAM	1IDO	Not applicable
JAK1	3EYG	0.306
JAK2	4D0X	0.628
JAK3	3LXK	0.972
IKK	4KIK	0.707
RAC1	5N6O	0.947
TYK2	4GIH	0.396
CSF1R	3LCO	0.653
Tubulin	1SA0	1.835
COX2	6COX	0,.853

Reference

1. Wang, X.; Li, W.; Ma, X.; Chu, Y.; Li, S.; Guo, J.; Jia, Y.; Zhou, S.; Zhu, Y.; Liu, C. Simultaneous determination of caffeic acid and its major pharmacologically active metabolites in rat plasma by LC-MS/MS and its application in pharmacokinetic study. Biomed Chromatogr. 2015. 29(4). 552-559.
2. Zhang, J.; Wu, X.; Qiu, J.; Zhang, L.; Zhang, Y.; Qiu, X.; Huang, Z.; Xu, W. Comprehensive Comparison on the Chemical Profile of Guang Chen Pi at Different Ripeness Stages Using Untargeted and Pseudotargeted Metabolomics. J Agr Food Chem. 2020. 68(31). 8483-8495.
3. Xu, L.L.; Xu, J.J.; Zhong, K.R.; Shang, Z.P.; Wang, F.; Wang, R.F.; Zhang, L.; Zhang, J.Y.; Liu, B. Analysis of Non-Volatile Chemical Constituents of Menthae Haplocalycis Herba by Ultra-High Performance Liquid Chromatography-High Resolution Mass Spectrometry. Molecules. 2017. 22(10).
4. Tang, J.; Dunshea, F.R.; Suleria, H.A.R. LC-ESI-QTOF/MS Characterization of Phenolic Compounds from Medicinal Plants (Hops and Juniper Berries) and Their Antioxidant Activity. Foods. 2019. 9(1).
